# One-pot ultrasound synthesis of polyaniline–ZnO and its synergistic effect on microbial activity

**DOI:** 10.1039/d5ra04461h

**Published:** 2025-10-10

**Authors:** Kalpana N. Handore, Sumit B. Sharma, Vasant V. Chabukswar

**Affiliations:** a Department of Engineering Science and Humanities, Marathwada Mitra Mandal College of Engineering Karvenagar Pune Pune 411052 Maharashtra India kalpanahandore@gmail.com (+91) 9975075760; b Department of Chemistry, Wadia Engineering College Bund Garden Road Pune India; c Department of Chemistry, Nowrosjee Wadia College Bund Garden Road Pune India

## Abstract

In this investigation, an eco-friendly ultrasonic-assisted green synthesis was employed to synthesize a polyaniline–zinc oxide (PANI–ZnO) nanocomposite with particle sizes ranging from 50 to 60 nm. Zinc oxide (ZnO) nanoparticles were synthesised using a low-cost coprecipitation approach, and the PANI–ZnO nanocomposite was formed *in situ* using an ultrasound, single-step oxidative polymerisation process. This approach promoted a uniform dispersion of the ZnO nanoparticles inside the polyaniline matrix, which improved the physicochemical interactions between the organic and inorganic phases. The structural, morphological, thermal, optical, and electrical properties of the nanocomposite were thoroughly investigated using ultraviolet-visible (UV-vis) spectroscopy, Fourier transform infrared (FTIR) spectroscopy, scanning electron microscopy (SEM), transmission electron microscopy (TEM), X-ray diffraction (XRD), thermogravimetric analysis (TGA), and electrical conductivity measurements. UV-vis and FTIR analyses showed the nanocomposite's effective production and interaction with ZnO and PANI functional groups. SEM scans revealed that the ZnO nanoparticles were uniformly dispersed throughout the PANI matrix, with no agglomeration. XRD patterns demonstrated that the crystalline character of ZnO was preserved within the composite, while TGA results indicated that the nanocomposite was more thermally stable than pure PANI. Electrical conductivity experiments revealed that the addition of ZnO altered charge transport capabilities, increasing the composite's suitability for electronic applications. Furthermore, the antibacterial activity of the PANI–ZnO nanocomposite was tested against *Escherichia coli* (*E. coli*), *Staphylococcus aureus* (*S. aureus*), and *Salmonella typhi* (*S. typhi*). The nanocomposite exhibited better antibacterial activity against *E. coli* and *S. aureus* but reduced activity against *S. typhi*. The observed antibacterial behaviour was due to the synergistic interaction between the ZnO nanoparticles and the PANI matrix, which resulted in improved disruption of bacterial cell membranes. Overall, the PANI–ZnO nanocomposite synthesised *via* this green, ultrasonic technique showed remarkable multifunctional capabilities, making it a feasible option for applications in antimicrobial materials, sensors, and electronic devices.

## Abstract 2

In this investigation, an eco-friendly ultrasonic-assisted green synthesis was employed to synthesize a polyaniline–zinc oxide (PANI–ZnO) nanocomposite with particle sizes ranging from 50 to 60 nm.

## Graphical abstract

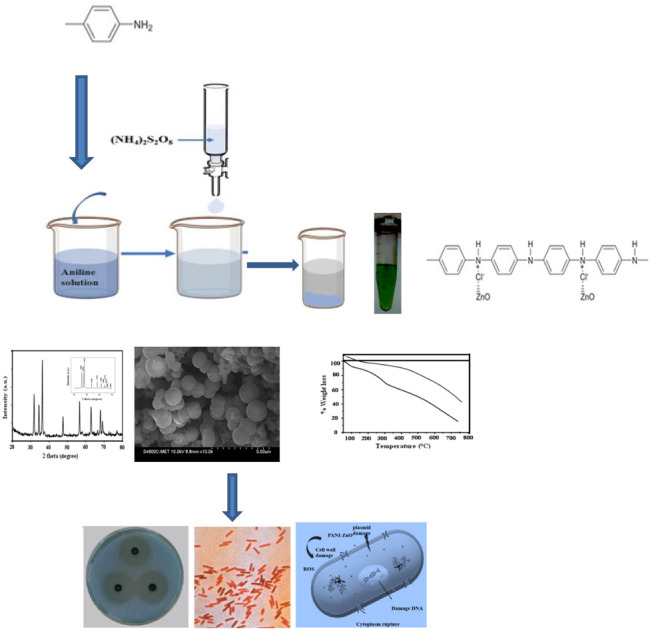

## 1. Introduction

Organic intrinsic conducting polymers, such as polyaniline, polyindole, polythiophene and polypyrrole, are excellent materials. In recent times, scientists and scholars have become increasingly interested in a wide range of promising polymers and their nanocomposites because of their superior electrical and good optical properties.^1,2^ Heterogeneous catalysis, lithium cells, biosensors and battery electrodes are just a few of their many applications.^3–5^ In order to enhance the electrical characteristics of polyaniline and its nanocomposites, recent work has concentrated on the environmentally friendly synthesis of nanocomposites and their possible applications in a variety of electronic devices.^6^

Transformation of bulk matter to nanoparticles leads to a change in its various properties. An increase in the surface area increases most of the properties like catenation and enhances the strength, heat, and mechanical, electronic and electrical properties. Nano-scale devices with engineered properties result in an improvement in the quality of materials that are useful in a variety of applications.^7–9^ ZnO shows unique electrical, mechanical and optical properties; therefore, it is used in applications in various fields. The small size of nanomaterials finds multiple applications in technical and medicinal fields. Zinc oxide nanoparticles are superior inorganic substances. They have applications in textiles, energy conservation, electronics, healthcare, cosmetics, semiconductors, environmentally friendly catalysis, and chemical sensing.^10–15^ The nontoxic and biocompatible nature of these nanoparticles makes them useful in biomedical applications such as targeted drug delivery, wound healing, and bioimaging, and they also have anticancer and anti-inflammatory properties.^16,17^ In the literature, various methods have been reported for the synthesis of PANI–ZnO, including *in situ* polymerization, electrochemical deposition, and physical mixing, as shown in Table 2, but recently, ultrasonic-aided synthesis has been used to prepare conducting nanocomposites as it gives excellent morphology and antibacterial activity.

The distinct diameter of nanometal oxides like SnO_2_, TiO_2_, Fe_2_O_3_, CuO, SiO_2_, and CeO_2_ make them suitable for use in solar cell applications.^18^ Compared to bulk materials, the prepared nano-ZnO and nanocomposites exhibit superior characteristics. Numerous methods of synthesis ZnO nanoparticles have been documented in the literature (Table 1).^19–23^ A few possible applications of nanocomposites include gas sensors, photoelectric applications, UV-emitting diodes, dye-sensitized solar cells, and biological sensors, as well as in the ceramic and paint industries.

**Table 1:** Reported ZnO nanoparticle synthesis methods and the resultant morphologies

Sr. no.	Method of synthesis	ZnO morphology
1	Microwave decomposition	Spherical shape^19^
2	Simple wet chemical route	Nano-size microflowers, dumbbell shape^20^
3	Deposition process	Rod-shaped^21^
4	Solvo-thermal method	Nanoflowers, nanorods, nanospheres^22^
5	Simple precipitation method	Nanoflakes^23^
6	Hydrothermal synthesis	Hexagonal prismatic rods, nanorods^24^
7	Microwave hydrothermal method	Mulberry-like^25^

**Table 2:** PANI–ZnO nanoparticle synthesis methods

Sr. no.	Synthesis method	PANI–ZnO morphology
1	*In situ* polymerization	ZnO nanoparticles are dispersed in aniline, and polymerization is initiated with an oxidizing agent. In this, PANI-wrapped ZnO particles are observed.^26^
2	Sol–gel method	ZnO preparation by sol–gel and incorporation of aniline for controlled synthesis. Fine ZnO particles embedded in PANI are observed.^27^
3	Electrochemical polymerization	Aniline is polymerized electrochemically in the presence of ZnO nanoparticles. It has a nanofibre and granular morphology.^28^
4	Mechanical milling	Physical mixing of PANI and ZnO powders is followed by heat treatment for better interaction. Less uniformity in particles.^29^
5	Hydrothermal synthesis	ZnO nanorods or nanowires coated or wrapped by PANI layers; hierarchical nanostructures. In this method, reported nanowires or nanorods are coated with PANI layers.^30^
6	Microwave-assisted synthesis	Uniform nanoparticles or nanofibers with smaller particle sizes are obtained due to fast nucleation. Morphology has a uniform distribution with nanofibers.^32^

Younas Sohail reported green-synthesized ZnO/CuO nanoparticles coated with PANI that showed significantly higher antifungal inhibition (∼77%) against *Aspergillus parasiticus* compared to bare nanoparticles.^31^ Charoensri *et al.*, in 2021, reported a PANI–ZnO composite with thermoplastic starch films that exhibited ∼76% and ∼72% antibacterial activity against *E. coli* and *S. aureus*, respectively.^33^ Singh *et al.*,^32^ in 2019, reported strong antibacterial effects of ZnO with a size less than 40 nm against *S. aureus* and *P. aeruginosa*. Khan *et al.*, in 2018, reported that 80–120 nm particles showed moderate antibacterial activity.^34^ According to these studies, an overall decrease in the size of polymer nanocomposites enhanced their antibacterial activity.

**Scheme 1:**
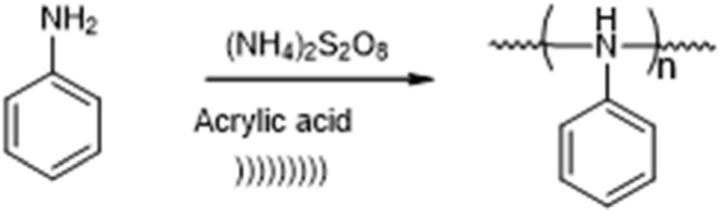
Ultrasound synthesis of polyaniline (PANI).

**Scheme 2:**
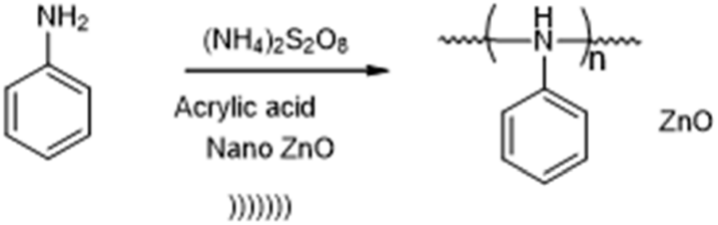
Ultrasound synthesis of polyaniline–ZnO (PANI–ZnO).

This report describes the ultrasound-assisted synthesis of polyaniline (Scheme 1) and polyaniline–ZnO (Scheme 2), which has been studied for its antibacterial activity and characterized by UV-vis, FT-IR, SEM, TEM, XRD and TGA.

## 2. Experimental

### 2.1 Materials and methods

Aniline and zinc acetate were distilled under low pressure. Analytical-grade chemicals were used, including acrylic acid, sodium hydroxide (Merck, India), and ammonium persulphate (APS). All analytical-grade solvents were distilled before usage. For synthesis, deionized water from the Millipore system was utilized. The technical specification of the ultrasonicator for synthesis is as follows: electric supply, 230 V; ultrasonic frequency, 33 kHz; ultrasonic power, 100 W.

### 2.2 Ultrasonic-assisted synthesis of polyaniline

Polyaniline was produced by the oxidative polymerization of aniline using an ultrasonic method.^19^ Double-distilled aniline (10 mL) and acrylic acid (0.5 mL) were added to a 100-mL round-bottom flask at 25 °C. Next, 25 mL of a 1 M APS solution was added dropwise gradually, and the reaction mixture was continuously stirred for two hours. After 30 minutes of stirring and 20 minutes of sonication, the solution gradually turned dark green and was kept at room temperature for an hour. The dark-green solution was then washed with DI water and dried in a vacuum oven at 60 °C for two hours in order to produce green-colored polyaniline.^8^

### 2.3 Ultrasonic-assisted *in situ* synthesis of the polyaniline–ZnO nanocomposite

100 mL of distilled water was used to dissolve 2.0 g of Zn(CH_3_COO)_2_·2H_2_O. The aniline is added dropwise to this solution. After adding a 1 M sodium hydroxide solution (10 mL) gradually and mixing vigorously for 20 minutes at room temperature (27 °C), the mixture was sonicated for an additional 20 minutes to get a precipitate. It was then dried in an oven at 70–80 °C.

## 3. Results and discussion

### 3.1 UV analysis

**Fig. 1:**
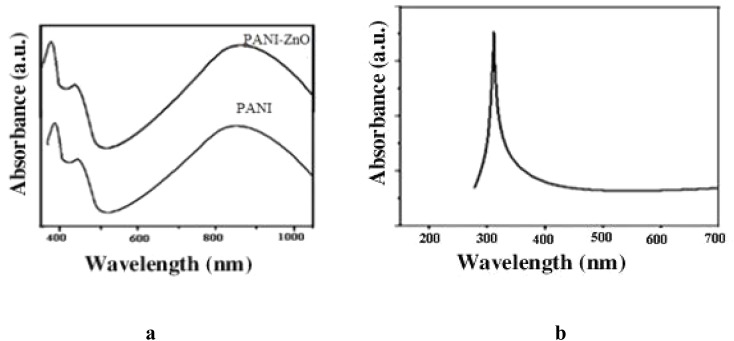
(a) UV-visible absorption spectra of (a) polyaniline and PANI–ZnO and (b) ZnO.

Fig. 1a and b display the optical absorption peaks of the ZnO nanoparticles, polyaniline, and polyaniline–ZnO nanocomposites. The three absorption bands at 325, 450 and 820 nm in Fig. 1a indicate that it is doped polyaniline. At 325 and 820 nm, polyaniline displays broad, sharp bands that correspond to the π–π* transitions of the benzenoid groups and the n–π* transitions, respectively. The spectrum of ZnO (Fig. 1b) exhibits a recognizable, single, sharp peak at 315 nm. The strong, acute peak suggests that the particles have a hexagonal wurtzite structure and are small in size. The prepared ZnO nanoparticle is in the pure form, as indicated by this prominent single peak. The polyaniline–ZnO nanocomposite's spectrum is shown in Fig. 1a; a notable redshift is observed between 820 and 835 nm and between 325 and 360 nm. The interaction between the polyaniline and ZnO hydroxyl groups causes the redshift.^23^

### 3.2 FT-IR analysis

**Fig. 2:**
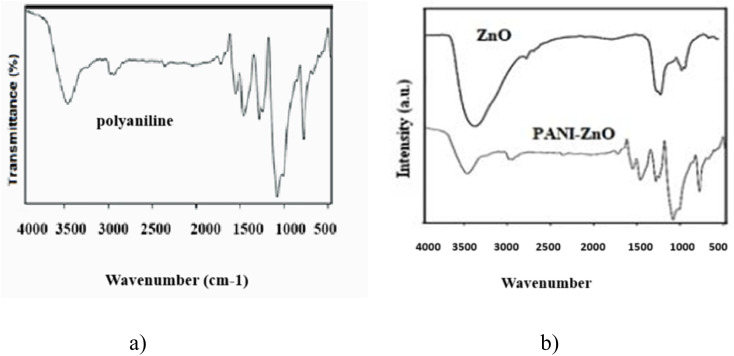
FT-IR spectra of (a) polyaniline and (b) ZnO and PANI–ZnO.

The FT-IR spectra of polyaniline, ZnO and the PANI–ZnO nanocomposite are shown in Fig. 2. The figure shows the major characteristic sharp peaks at 3450, 3055, 1585, and 820 cm^−1^, which are similar to those of the standard PANI reported in the literature. The presence of quinonoid is indicated by the characteristic peak at 1579 cm^−1^, and benzenoid rings are responsible for the peak at 1490 cm^−1^. The stretching vibrations of the C–C ring, both symmetric and asymmetric, are responsible for the observed peaks at 1562 cm^−1^ and 1496 cm^−1^, respectively. The peak located at 2870 cm^−1^ is indicative of C–H stretching (Fig. 2a). The FT-IR spectrum of the ZnO nanoparticle (Fig. 2b) reveals a peak at 3480 cm^−1^, which is caused by the –OH stretching. The polyaniline–ZnO nanocomposite's FT-IR spectrum shows the presence of zinc oxide with three new absorption peaks at 3500 cm^−1^, 860 cm^−1^, and 740 cm^−1^. The peak at 3220 cm^−1^ is attributed to N–H stretching, while the peaks at 850 cm^−1^ and 740 cm^−1^ are associated with Zn–OH and Zn–O–Zn bonds, respectively. The broad peak at 3500 cm^−1^ indicates the presence of the –OH group, indicating strong interactions between ZnO and polyaniline (Fig. 2b).

### 3.3 XRD analysis

**Fig. 3:**
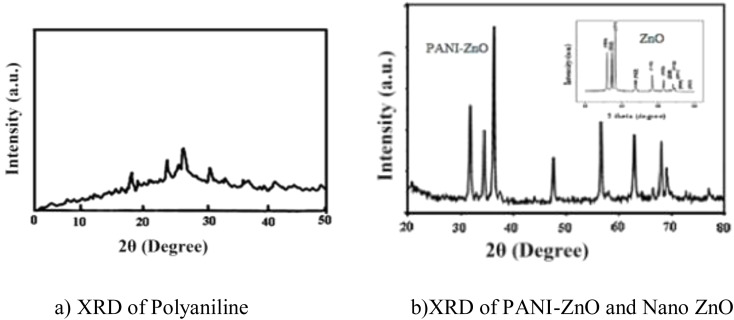
XRD (a) polyaniline (b) PANI-ZnO and nano ZnO.

Fig. 3 displays the XRD patterns of ZnO, polyaniline and the PANI–ZnO nanocomposite. The XRD pattern demonstrates that the prepared polyaniline has a crystalline nature, and no extra peaks are observed, indicating the purity of PANI as shown in (Fig. 3a). Fig. 3b presents the XRD patterns of ZnO and PANI–ZnO, which show reflection planes resembling hexagonal ZnO with a 50–60 nm size, according to Scherer's equation. The wurtzite hexagonal structure of ZnO matches with JCPDS-36-1451 in the literature.^20^ The XRD pattern of the nanocomposite shows sharp and well-defined diffraction peaks, which indicate the crystallinity of the material.

### 3.4 Morphological studies using scanning electron microscopy (SEM)

**Fig. 4:**
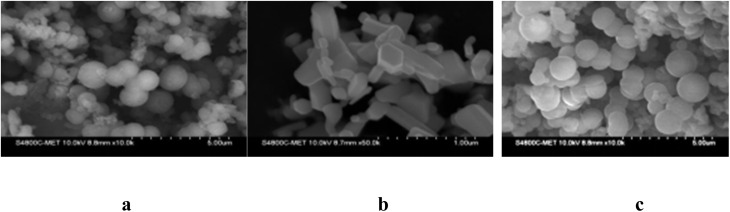
SEM images of (a) polyaniline, (b) the ZnO nanoparticles and (c) polyaniline–ZnO.

Fig. 4a shows how acrylic acid affects the structural morphology of polyaniline and also shows how the diameter of polyaniline is impacted by the type and size of dopants used in the polymerization process. Polyaniline is synthesized with ultrasound assistance; it exhibits a clear, well-defined circular morphology. These particles aggregate into clusters with an average diameter of 60 nm. The polyaniline produced is of the best quality with an excellent structural morphology.

Fig. 4b shows the SEM image of ZnO nanoparticle, which has a well-defined hexagonal morphology and is 50 nm in diameter, while Fig. 4c shows SEM image polyaniline–ZnO having agglomeration. The nanocomposite shows an excellent spherical morphology, and ZnO is encapsulated by the polyaniline polymer, with synergistic interactions observed between them.

### 3.5 Morphological studies of ZnO and PANI–ZnO using TEM

**Fig. 5:**
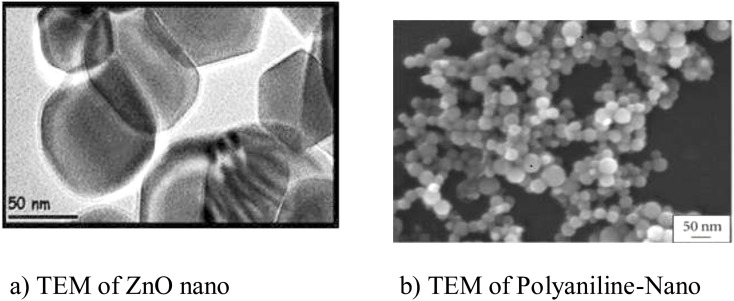
(a) TEM images of (a) ZnO nanoparticle (b) polyaniline–ZnO.

The electron micrographs of the synthesized ZnO and PANI–ZnO are shown in Fig. 5. The TEM images show details of the morphology of ZnO and nanocomposite particles, *i.e.* size and shape. The TEM image of ZnO clearly shows the wurtzite structure of ZnO as the particles are hexagonal in shape, which supports the SEM results (Fig. 5a). The TEM image of PANI–ZnO shows a spherical morphology with ZnO embedded in the polymer matrix (Fig. 5b).

### 3.6 Thermal analysis

**Fig. 6:**
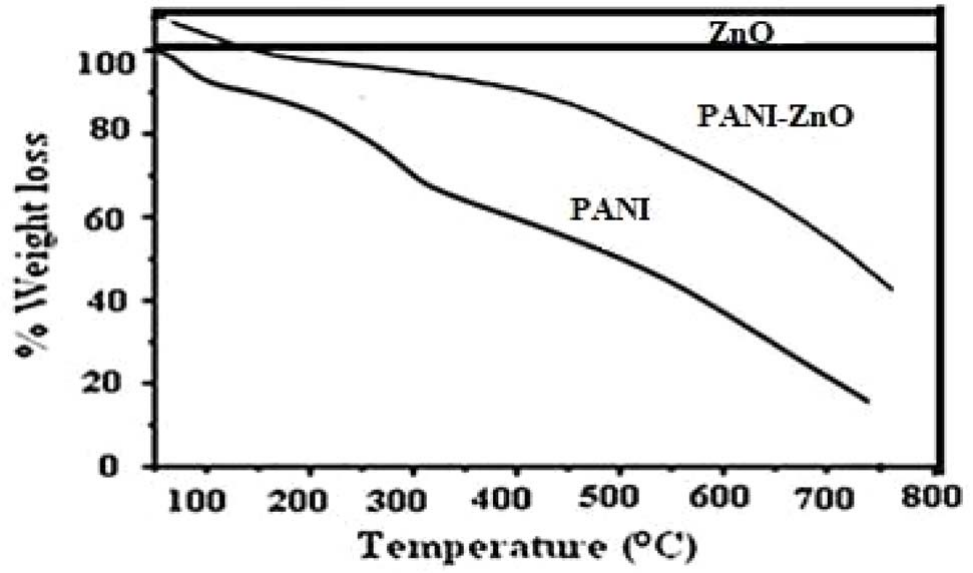
TGA analysis of PANI, PANI–ZnO and ZnO.

Fig. 6a shows the thermograms of polyaniline, nano-ZnO and the polyaniline–ZnO nanocomposite. Fig. 5a shows two decomposition steps; initially, fragmentation takes place between 90 °C and 120 °C, with a weight loss of approximately 5%, which is indicative of water loss. The second weight loss happens between 120 °C and 450 °C, and it might be brought on by the organic moiety breaking down. The polyaniline–ZnO nanocomposite exhibits initial degradation at 100 °C, followed by a 4% weight loss, and a 30% weight loss at 580 °C. TGA shows that the nanocomposite is more stable than polyaniline, and the stability in the nanocomposite is due to the ZnO nanoparticles. It is clear that ZnO is more thermally stable than the nanocomposite.

### 3.7 Electro-conductivity

The electrical conductance of the synthesized polyaniline and ZnO nanocomposite was measured by the four-probe method. It is observed that the conductivity of the material increases with a rise in temperature. The ZnO nanoparticles may have been incorporated into polyaniline, as evident by the electrical conductivity values of 3.6 S cm^−1^ and 3.4 × 10^−2^ S cm^−1^ for polyaniline and the polyaniline nanocomposite, respectively. The conductivity of the nanocomposite is low when compared to that of pure polyaniline.

### 3.8 Antibacterial activity

Using the well diffusion method, the antimicrobial activity of polyaniline–ZnO was investigated on bacterial strains classified as Gram-positive and Gram-negative.^21^ The samples were exposed to UV to disinfect them before any antibacterial action. It was noted that the ultrasound-synthesized PANI–ZnO nanocomposite utilized was a more potent antibacterial agent against *E. coli* than against *S. typhi*.

**Fig. 7:**
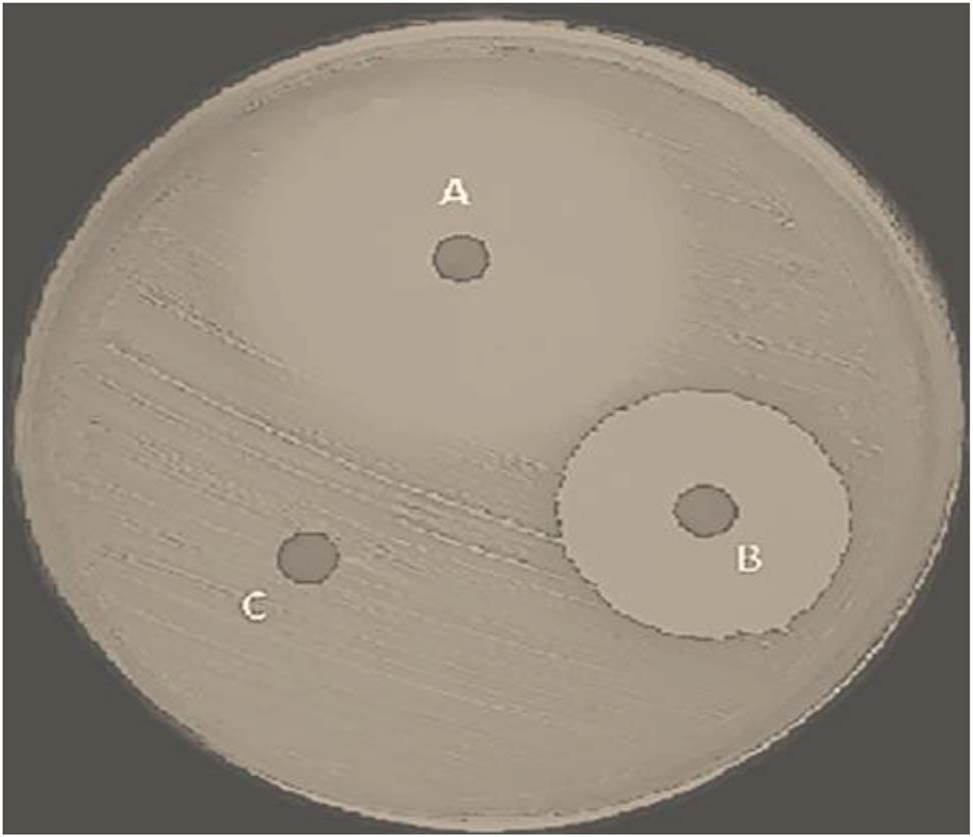
Comparision of antibacterial activity of (A) PANI-ZnO, (B) ZnO nanoparticle and (C) PANI against *E. coli*.

Nanocomposites were screened for their antibacterial activity against the Gram-positive bacterial strains such as *Staphylococcus aureus* (NCIM 8903) and Gram-negative strains *Escherichia coli* (NCIM 8902) and *S. typhi* (NCIM 8901). To measure the antibacterial activity at different concentration levels, PANI–ZnO was dissolved in DMSO and prepared in concentrations of 5, 10, 15, 20, 25, 40, 80, 100, and 200 μg mL^−1^. Tables 3 and 4 report the results of the inhibition zone measured in order to assess the antimicrobial activity.^20,21^ Ciprofloxacin at a concentration of 10 μg mL^−1^ was used as a standard positive control. Fig. 7 shows comparision of antibacterial actvity of PANI, ZnO nanoparticle and PANI-ZnO. ZnO nanoparticles in PANI improve its antibacterial activity compared to the material alone as shown in Fig. 7A this is due to synergestic effect of PANI and ZnO. The synthesized PANI Fig. 7C does not show antibacterial activity; this may be because the long-chain structure of PANI is unable to penetrate the bacterial cell. However, the PANI–ZnO nanocomposite shows an inhibition zone, clearly indicating the biocidal effect of ZnO, which indicates the disruption of the membrane with a high rate of generation of oxygen species and leads to the death of bacteria.

**Table 3:** Effect of the concentration of PANI–ZnO on antibacterial activity

Sr. no.	PANI–ZnO (mg mL^−1^)	Zone of inhibition (mm) for *E. coli*	Zone of inhibition (mm) for *S. aureus*
1	0	0	0
2	1	9	10
3	2	14	15
4	3	15	20
5	4	16	20

**Table 4:** Antimicrobial activity of the nanocomposite compared with a standard antibiotic

Sr. no.	Compound	*S. aureus*	*E. coli*	*S. typhi*
1	Polyaniline–ZnO	20	15	7
2	Ciprofloxacin	23	20	10

**Fig. 8:**
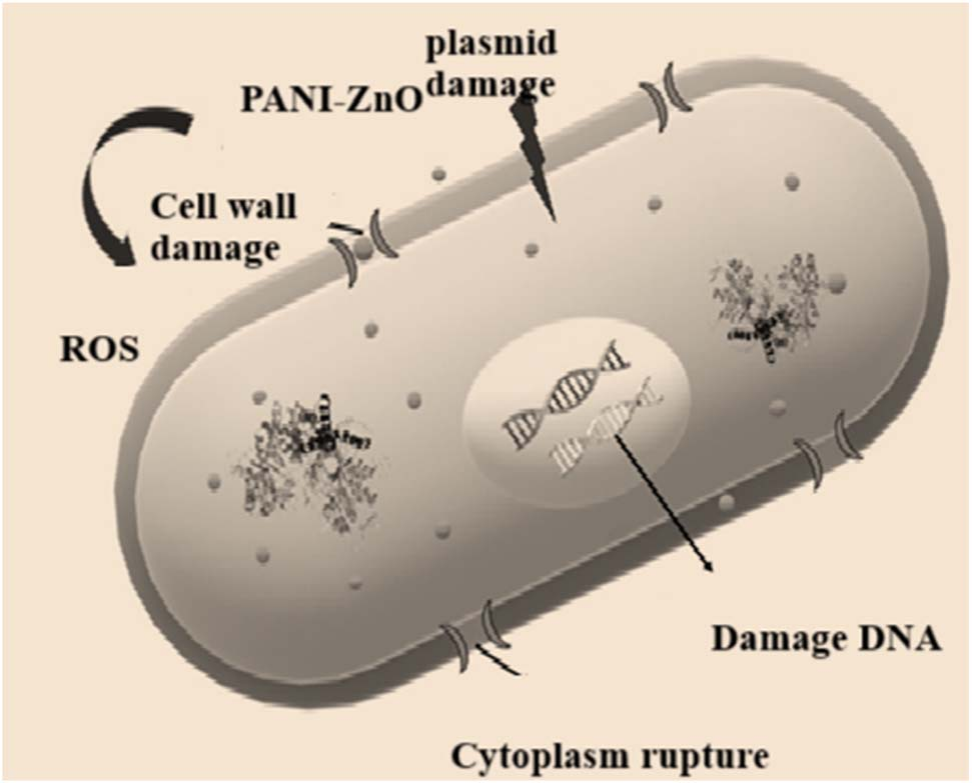
Mechanism of the antibacterial activity of PANI–ZnO.

Fig. 8 illustrates the mechanism of the antibacterial activity of PANI–ZnO. As PANI–ZnO come in contact with the cell wall it releases of Zn^+2^ ions. ZnO nanoparticles are embedded in a polyaniline matrix, enhances its interaction with bacterial cell walls, leading to damage of cell wall and plasmid also it damages DNA and ultimately cell death.

At high concentrations of the nanocomposite (PANI–ZnO), nanoparticles aggregate, which reduces the surface area and decreases the antibacterial activity. Tsao, Chun Wen *et al.* reported that the concentration of ZnO in the PANI–ZnO composite directly affects its antibacterial activity.^35^ At the lowest concentration, *i.e.* <10 wt% of ZnO in PANI–ZnO, it shows the lowest antibacterial activity; this is due to the lack of active ZnO sites. By contrast, at moderate concentrations, *i.e.* 20–40% of ZnO in the polymer, the composite shows optimum activity as it balances dispersion and reactivity. Greater concentrations (>50 wt%) decrease the antibacterial activity due to agglomeration of particles, which reduces the active surface area and antimicrobial activity.

It is observed from the above table that the antibacterial activity depends on the concentration. As the concentration of the nanocomposite increases, the antibacterial activity also increases. The antibacterial activity is the maximum at a concentration of 3 mg mL^−1^ but the lowest at 1 mg mL^−1^ (Table 3, entries 3 and 4). The antibacterial activity is generally higher against *S. aureus* (Gram-positive) than *E. coli* (Gram-negative) due to differences in their cell wall structure. A further increase in the concentration does not lead to any appreciable change (Table 3, entry 4).

The above table shows that significant antibacterial activity was shown by the PANI–ZnO nanocomposite, especially against *S. aureus* (20 mm zone of inhibition), which was comparable to the efficacy of the common antibiotic ciprofloxacin (23 mm), while showing reduced activity against *S. typhi*.

## 4. Conclusion

A polyaniline–ZnO nanocomposite was prepared under ultrasonic conditions using an oxidant in a single step through the *in situ* polymerization method. Ultrasound synthesis enhanced polymerization and avoided the aggregation of nanoparticles. The ZnO and nanocomposite preparation were verified using various spectral techniques. The FT-IR and UV-vis analysis results supported the hexagonal wurtzite structural morphology of ZnO, which was distributed uniformly throughout the matrix of polyaniline. The ZnO nanoparticles and polyaniline exhibited chemical interactions, as confirmed by SEM. The nanocomposite showed excellent antimicrobial properties against *S. Aureus* and *E. coli* strains, but was less active towards *S. typhi*. The greater antibacterial activity was due to the synthesis of PANI–ZnO using the ultrasound technique, which, besides being an eco-friendly method, also gives well-dispersed ZnO in polyaniline. The ultrasound method of synthesis also controls the morphology of nanoparticles. Due to their small size and higher surface area-to-volume ratio, more efficient interactions with bacterial cells were possible. The spectral studies also justified the synergistic interaction between PANI and ZnO in the nanocomposite, which enhanced each material's unique qualities and improved antibacterial activity. Its efficacy was attributed to the nanocomposite's capacity to rupture bacterial membranes and produce reactive oxygen species (ROS). The performance of the PANI–ZnO nanocomposite was also impacted by the overall structure, the size and shape of the nanoparticles, and the ratio of PANI to ZnO.

## Conflicts of interest

There are no conflicts to declare by any of the author.

## Data Availability

The authors declare that the data supporting the findings of this study are available within the paper. All spectral data, figures and tables are included and available within the paper.
